# Risk estimation for postoperative nausea and vomiting: development and validation of a nomogram based on point-of-care gastric ultrasound

**DOI:** 10.1186/s12871-023-02345-0

**Published:** 2023-11-30

**Authors:** Huohu Zhong, Yingchao Liu, Piaopiao Liu, Zecheng Wang, Xihua Lian, Zhirong Xu, Ruopu Xu, Shanshan Su, Guorong Lyu, Zhenhong Xu

**Affiliations:** 1 Department of Ultrasound Medicine, The Second Affiliated Hospital of Fujian Medical University, Quanzhou, China; 2 Department of Anesthesiology, The Second Affiliated Hospital of Fujian Medical University, Quanzhou, China; 3 Department of Pathology and Biomedical Science, University of Otago, Christchurch, New Zealand; 4 Department of Emergency, The Second Affiliated Hospital of Fujian Medical University, Quanzhou, China; 5 Collaborative Innovation Center for Maternal and Infant Health Service Application Technology of Education Ministry, Quanzhou Medical College, Quanzhou, China

**Keywords:** Postoperative nausea, Vomiting, Point-of-care ultrasound, Enhanced recovery after surgery, Nomogram

## Abstract

**Background:**

We aimed to develop a nomogram that can be combined with point-of-care gastric ultrasound and utilised to predict postoperative nausea and vomiting (PONV) in adult patients after emergency surgery.

**Methods:**

Imaging and clinical data of 236 adult patients undergoing emergency surgery in a university hospital between April 2022 and February 2023 were prospectively collected. Patients were divided into a training cohort (*n* = 177) and a verification cohort (*n* = 59) in a ratio of 3:1, according to a random number table. After univariate analysis and multivariate logistic regression analysis of the training cohort, independent risk factors for PONV were screened to develop the nomogram model. The receiver operating characteristic curve, calibration curve, decision curve analysis (DCA) and clinical impact curve (CIC) were used to evaluate the prediction efficiency, accuracy, and clinical practicability of the model.

**Results:**

Univariate analysis and multivariate logistic regression analysis showed that female sex, history of PONV, history of migraine and gastric cross-sectional area were independent risk factors for PONV. These four independent risk factors were utilised to construct the nomogram model, which achieved significant concordance indices of 0.832 (95% confidence interval [CI], 0.771–0.893) and 0.827 (95% CI, 0.722–0.932) for predicting PONV in the training and validation cohorts, respectively. The nomogram also had well-fitted calibration curves. DCA and CIC indicated that the nomogram had great clinical practicability.

**Conclusions:**

This study demonstrated the prediction efficacy, differentiation, and clinical practicability of a nomogram for predicting PONV. This nomogram may serve as an intuitive and visual guide for rapid risk assessment in patients with PONV before emergency surgery.

## Background

Postoperative nausea and vomiting (PONV) is a common postoperative adverse reaction occurring within 24 h after surgery [1]. PONV is not only a painful postoperative recovery experience but can also cause dehydration, electrolyte imbalance, aspiration pneumonia, pneumothorax, hypoxia, oesophageal rupture, intracranial pressure, and a series of other complications, resulting in fatigue, anxiety, accidental hospitalisation, readmission, or even mortality [1, 2]. The prevention of PONV is very important for enhanced recovery after surgery [3–5].

Currently, prophylactic antiemetics are mainly used to reduce the incidence of PONV, and 5-HT3 receptor antagonists combined with 4 or 8 mg dexamethasone are the most widely used regimens for the prevention thereof [6]. However, Medikonda et al. [7] indicated that preoperative and postoperative combined use of dexamethasone can increase the risk of postoperative wound infection and lead to a series of side effects including immunosuppression, insulin resistance, hyperglycaemia, and venous thromboembolism, which have a negative impact on prognosis; the side effects of preoperative combined use are more obvious. Overusing 5-HT3 receptor antagonists can lead to headaches, intractable constipation, aminotransferase elevation, and a prolonged QT interval [8]. Therefore, accurate prediction of high-risk patients is particularly important in the prevention and treatment of PONV.

At present, Apfel [9] and Koivuranta [10] scores are the most widely used methods for assessing the risk of PONV in patients; however, researchers from different countries have reported that these scores are not very effective in predicting PONV in their own populations [11–13]. Cozza et al. [14] stated that these scores only consider clinical parameters and cannot accurately predict PONV. Roulin et al. [15] reported that patients undergoing emergency surgery were usually unable to perform adequate preoperative intestinal preparation due to insufficient preparation time and a more critical condition; as a result, the incidence of postoperative complications was higher than that of patients undergoing elective surgery. In recent years, point-of-care gastric ultrasound has been widely used in the perioperative period as an innovative technology due to its advantages of being non-invasive and not using radiation; furthermore, it can provide valuable information regarding the type and volume of stomach contents [16].

The objective of this study was to extensively evaluate potential risk factors for PONV, construct a PONV prediction model and develop a nomogram for visual and practical application. To our knowledge, this is the first study to construct a nomogram combined with point-of-care gastric ultrasound as an innovative technology to visually predict PONV risk.

## Materials and methods

### Patients

A total of 236 adult patients undergoing emergency surgery were prospectively and continuously included from April 2022 to February 2023 at the Second Affiliated Hospital of Fujian Medical University. We included patients meeting the following criteria: (1) non-pregnant adults undergoing emergency surgery; (2) American Society of Anesthesiologists grade I–II; (3) patients without complications such as hypertension, coronary heart disease and diabetes before surgery; and (4) patients without other severe systemic disease. We excluded patients undergoing chemoradiotherapy before surgery, patients with preoperative pyloric obstruction, patients with hypoproteinaemia and anaemia before surgery, patients undergoing total gastrectomy or exploratory laparotomy and patients admitted to the intensive care unit after surgery. This study was approved by the Ethics Committee of the Second Affiliated Hospital of Fujian Medical University and performed in accordance with the ethical standards as laid down in the 1964 Declaration of Helsinki and its later amendments.

### *Preoperative* ultrasound examination

**Fig. 1:**
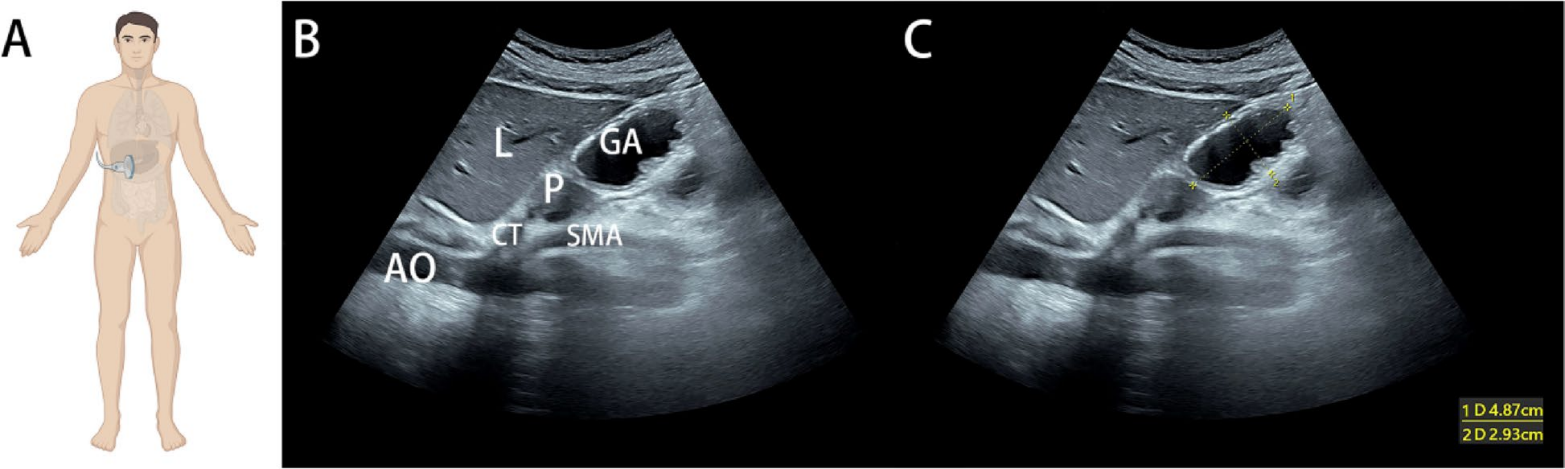
**A** Schematic diagram of ultrasonic probe placement. **B** Ultrasound examination of the gastric antrum. GA, gastric antrum; L, liver; P, pancreas; SMA, superior mesenteric artery; CT, coeliac trunk; AO, aorta. **C** The CSA measurement is based on the anteroposterior diameter and craniocaudal diameter. CSA, cross-sectional area

A colour Doppler ultrasound diagnostic apparatus (Mindray M6, Shenzhen, China) with a convex array probe (frequency 2–5 MHz) was used to select the abdominal system imaging mode to detect the gastric antrum of the patient. The patient was asked to lay in the right decubitus position. Point-of-care gastric ultrasound is more effective in detecting gastric contents at the right decubitus position because the fluid and solid fluid mixture flow with gravity to the antrum, while the gas collects upward at the bottom of the stomach [17–19]. At this point, images of the gastric antrum could be continuously observed through the sagittal plane of the upper abdomen, and the probe was then placed in the subxiphoid region of the patient. The gastric antrum could be explored through the sagittal section, and the standard section was positioned behind the left liver and in front of the abdominal aorta. After the standard section was determined, the anteroposterior diameter (AP) and craniocaudal diameter (CC) of the antrum were measured, and the images were retained (Fig. 1). The formula for estimating the cross-sectional area (CSA) was as follows: [20].

$${\mathrm{CSA }(\mathrm{cm}}^{2})=(\mathrm{AP }\times \mathrm{ CC }\times\uppi )/4$$

CSA was measured three times for each patient and averaged. The ultrasound examination was completed by a highly trained sonographer, and the obtained ultrasonogram was submitted to a sonographer with the title of associate senior or above for review.

### Data *collection*

The outcome index of this study was whether the patient had PONV; this was determined via follow-up with patients in the ward on the second day after surgery. The diagnostic criterion for PONV was the occurrence of postoperative nausea and/or vomiting within 24 h after surgery. The diagnosis of postoperative vomiting was mainly obtained through follow-up with the patient, the patient’s family, and the assigned nurse.

The diagnosis of postoperative nausea was obtained using a visual analogue score [21]; the scale plate was approximately 10-cm long and marked with a zero at one end and 10 at the other. Zero was classified as no nausea, and 10 was classified as intolerable nausea. Patients were asked to score the degree of nausea within 24 h after surgery, and postoperative nausea was defined as a score > 2.

The patient’s clinical and surgical data were recorded by accessing the electronic medical records system and anaesthesia system. The recorded items included patient sex, age, smoking history, alcohol history, PONV history, motion sickness history, migraine history, body mass index (BMI), duration of surgery, surgical position, mode of anaesthesia, type of inhaled anaesthetics, postoperative patient-controlled analgesia, intraoperative sufentanil dosage, duration of anaesthesia, and intraoperative use of neostigmine and glucocorticoids.

### *Statistical* analysis

SPSS version 27.0.1.0 (SPSS Inc., Chicago, IL) and R-language 4.2.2 (R Foundation for Statistical Computing, Vienna,Austria) were used to analyse the data. The intraclass correlation coefficient (ICC) was used to assess the consistency of CSA between the same physician and other similarly qualified physicians. All patients were divided into the training cohort (*n* = 177) and the verification cohort (*n* = 59) in a ratio of 3:1, according to a random number table. Measurement data conforming to a normal distribution were expressed as the mean ± standard deviation (x̄ ± *s*), and quantitative data between the two groups were compared using an independent sample t-test. Non-normally distributed data were expressed as the median (interquartile range), and the Mann–Whitney U-test was used for comparisons between the two groups. Enumeration data were expressed as constituent ratios, and the chi-squared test was used to compare differences between the two groups.

The significance of each variable for PONV in the training cohort was evaluated by univariate logistic regression analysis. Variables with statistically significant differences in univariate logistic regression analysis were included in multivariate logistic regression analysis to identify independent risk factors related to the occurrence of PONV. The rms package of R version 4.2.2 was used to build a nomogram to predict PONV occurrence. The predictive performance of the nomogram was measured by the concordance index, and 1,000 bootstrap samples were drawn to decrease the overfit bias. For the application of the model, the probability of PONV in each patient was calculated based on the nomogram. The receiver operating characteristic (ROC) curve was used to calculate the optimal threshold, which was determined by the maximum Youden index (i.e., sensitivity + specificity – 1), and the accuracy of the optimal threshold was evaluated with the sensitivity, specificity, predicted value and likelihood ratio. The calibration curve, decision curve analysis (DCA) and clinical impact curve (CIC) were used to further evaluate the predictive efficacy, accuracy and clinical practicability of the model.

### Patient and public involvement

This study included interviews with patients undergoing emergency surgery at our hospital.

## Results

### Basic characteristics of patients

Among the 236 adult patients who underwent emergency surgery in our hospital, 110 were males and 126 were females; the age range was 18–90 (mean, 46.03 ± 17.24) years (Table 1). In total, 87 (36.86%) patients had PONV and 149 (63.14%) patients had no PONV. The ICCs measured by the same physician and different physicians with the same qualifications for CSA were 0.979 (95% confidence interval [CI], 0.949–0.992) and 0.967 (95% CI, 0.918–0.987), respectively. The ICCs showed good consistency and reproducibility for CSA measured by the same physician and different physicians with the same qualifications.

**Table 1:** Participant characteristics in the training and validating cohorts

	Cohorts		
Variable	Training (*n* = 177)	Validating (*n* = 59)	*P* Value
Age [mean ± s]	45.34 ± 17.54	48.02 ± 16.31	0.378
Sex [n (%)]			0.183
Male	79 (44.63%)	31 (52.54%)	
Female	98 (55.37%)	28 (47.46%)	
BMI [mean ± s]	22.64 ± 3.65	22.61 ± 3.59	0.711
Smoking history [n (%)]			0.336
Yes	50 (28.25%)	19 (32.20%)	
No	127 (71.75%)	40 (67.80%)	
Alcohol [n (%)]			0.245
Yes	44 (24.86%)	18 (30.51%)	
No	133 (75.14%)	41 (69.49%)	
History of Motion sickness [n (%)]			0.471
Yes	45 (25.42%)	14 (23.73%)	
No	132 (74.58%)	45 (76.27%)	
History of PONV [n (%)]			0.418
Yes	26 (14.69%)	7 (11.86%)	
No	151 (85.31%)	52 (88.14%)	
History of Migraine [n (%)]			0.459
Yes	42 (23.73%)	15 (25.42%)	
No	135 (76.27%)	44 (74.58%)	
CSA [mean ± s]	5.47 ± 2.66	5.39 ± 2.35	0.518
Surgery type [n (%)]			** < 0.001**
Otolaryngological	11 (6.22%)	7 (11.86%)	
Gynaecological	46 (25.99%)	15 (25.42%)	
Open general	36 (20.34%)	8 (13.56%)	
Laparoscopic general	50 (28.25%)	18 (30.51%)	
Orthopaedic	19 (10.73%)	2 (3.39%)	
Urologic	15 (8.47%)	9 (15.26%)	
Duration of surgery [n (%)]			0.180
≤ 60 min	70 (39.55%)	28 (47.46%)	
> 60 min	107 (60.45%)	31 (52.54%)	
Operative position [n (%)]			0.443
Supine (including lithotomy)	156 (88.14%)	51 (86.44%)	
Non-supine position	21 (11.86%)	8 (13.56%)	
Duration of anaesthesia [n (%)]			0.411
≤ 90 min	88 (49.72%)	31 (52.54%)	
> 90 min	89 (50.28%)	28 (47.46%)	
Anaesthesia method [n (%)]			0.580
Intravenous-inhalation combined	157 (88.70%)	55 (93.22%)	
Intravenous	12 (6.78%)	2 (3.39%)	
Combined spinal and epidural	8 (4.52%)	2 (3.39%)	
Dosage of sufentanil used intraoperatively [mean ± s]	31.02 ± 15.91	29.15 ± 14.30	0.398
Dexmedetomidine used intraoperatively [n (%)]			0.537
Yes	125 (70.62%)	42 (71.19)	
No	52 (29.38%)	17 (28.81%)	
Neostigmine used intraoperatively [n (%)]			0.166
Yes	118 (66.67%)	44 (74.58%)	
No	59 (33.33%)	15 (25.42%)	
Glucocorticoid used intraoperatively [n (%)]			0.491
Yes	28 (15.82%)	10 (16.95%)	
No	149 (84.12%)	49 (83.05%)	
PCA used after surgery [n (%)]			0.075
Yes	84 (47.46%)	21 (35.59%)	
No	93 (52.54%)	38 (64.41%)	
Postoperative nausea and vomiting [n (%)]			0.172
Yes	65 (36.72%)	17 (28.81%)	
No	112 (63.28%)	42 (71.19%)	

*BMI* Body mass index, *CSA* Gastric cross-sectional area, *PCA* Patient-controlled analgesia, *PONV* Postoperative nausea and vomiting

### Development and validation of a PONV nomogram

Univariate logistic regression analysis showed that age, female sex, previous history of smoking, history of alcohol, history of motion sickness, history of migraine, history of PONV, CSA and absence of dexmedetomidine during surgery were risk factors for PONV (Table 2). The results of multivariate logistic regression analysis showed that sex, history of PONV, history of migraine and CSA were independent risk factors for PONV (Table 3). The results showed that female patients undergoing emergency surgery had a 6.329 times higher risk of developing PONV compared with male patients. Patients with a history of previous PONV and a history of migraine had a 6.072 times and 2.500 times increased risk of developing PONV, respectively, compared with to patients without such medical histories. Furthermore, for each 1 cm^2 increase in the measured gastric antrum cross-sectional area (CSA) using bedside ultrasound examination in preoperative emergency patients, the risk of PONV occurrence increased by 1.199 times.

**Table 2:** Univariate logistic regression analysis based on the training cohort

Variable	β Value	OR (95% CI)	*P* Value
Age, year	-0.21	0.979 (0.961–0.998)	**0.027**
Sex, male or female	-1.883	0.152 (0.073–0.316)	** < 0.001**
BMI, kg/m^2^	-0.72	0.930 (0.852–1.015)	0.104
History of smoking, yes or no	-1.453	0.234 (0.102–0.538)	** < 0.001**
History of alcohol, yes or no	-1.216	0.296 (0.128–0.666)	**0.005**
History of motion sickness, yes or no	0.807	2.240 (1.124–4.465)	**0.022**
History of PONV, yes or no	2.061	7.852 (2.957–20.852)	** < 0.001**
History of migraine, yes or no	1.525	4.595 (2.205–9.575)	** < 0.001**
CSA, cm^2^	0.245	1.278 (1.125–1.453)	** < 0.001**
Surgery type			
Otolaryngological			0.059
Gynaecological	0.647	1.909 (0.491–7.422)	0.351
Open general	-1.050	0.350 (0.077–1.583)	0.173
Laparoscopic general	0.154	1.167 (0.302–4.512)	0.823
Orthopaedic	-0.214	0.808 (0.169–3.858)	0.789
Urologic	-0.134	0.875 (0.171–4.472)	0.873
Duration of surgery > 60 min, yes or no	-0.232	0.793 (0.425–1.477)	0.465
Operative position, Supine position (including lithotomy position) or no	-0.693	0.500 (0.174–1.436)	0.198
Duration of anaesthesia > 90 min, yes or no	-0.164	0.849 (0.460–1.565)	0.600
Anaesthesia method			
Intravenous-inhalation combined			0.522
Intravenous	0.618	1.856 (0.483–7.127)	0.368
Combined spinal and epidural	0.001	1.000 (0.127–7.893)	1.000
Dosage of sufentanil used intraoperatively, mg	0.004	1.004 (0.985–1.024)	0.666
Dexmedetomidine used intraoperatively, yes or no	-0.677	0.508 (0.262–0.984)	**0.045**
Neostigmine used intraoperatively, yes or no	-0.145	0.865 (0.454–1.648)	0.659
Glucocorticoid used intraoperatively, yes or no	0.480	1.617 (0.715–3.654)	0.248
PCA used after surgery, yes or no	0.279	1.321 (0.714–2.444)	0.374

*BMI* Body mass index, *CI* Confidence interval, *CSA* Gastric cross-sectional area, *OR* Odds ratio, *PCA* Patient-controlled analgesia, *PONV* Postoperative nausea and vomiting

**Table 3:** Multivariate logistic regression analysis based on the training cohort

Variable	β Value	OR (95% CI)	*P* Value
Female	1.848	6.329 (2.740–14.706)	< 0.001
History of PONV	1.804	6.072 (1.923–19.175)	0.002
History of migraine	0.916	2.500 (1.067–5.856)	0.035
CSA	0.182	1.199 (1.037–1.386)	0.014

*CI* Confidence interval, *CSA* Gastric cross-sectional area, *OR* Odds ratio, *PONV* Postoperative nausea and vomiting

**Fig. 2:**
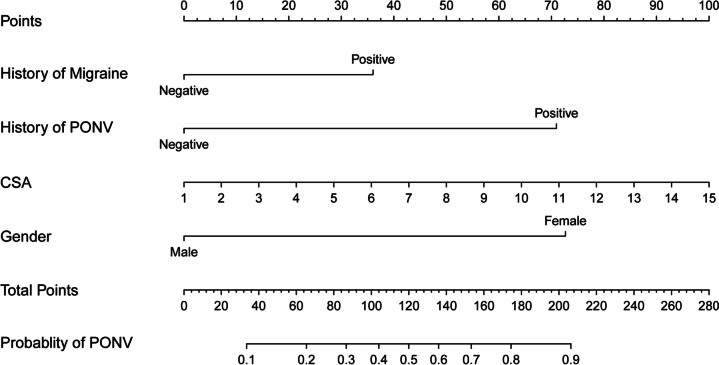
Nomogram to estimate the risk of PONV in patients undergoing emergency surgery

**Fig. 3:**
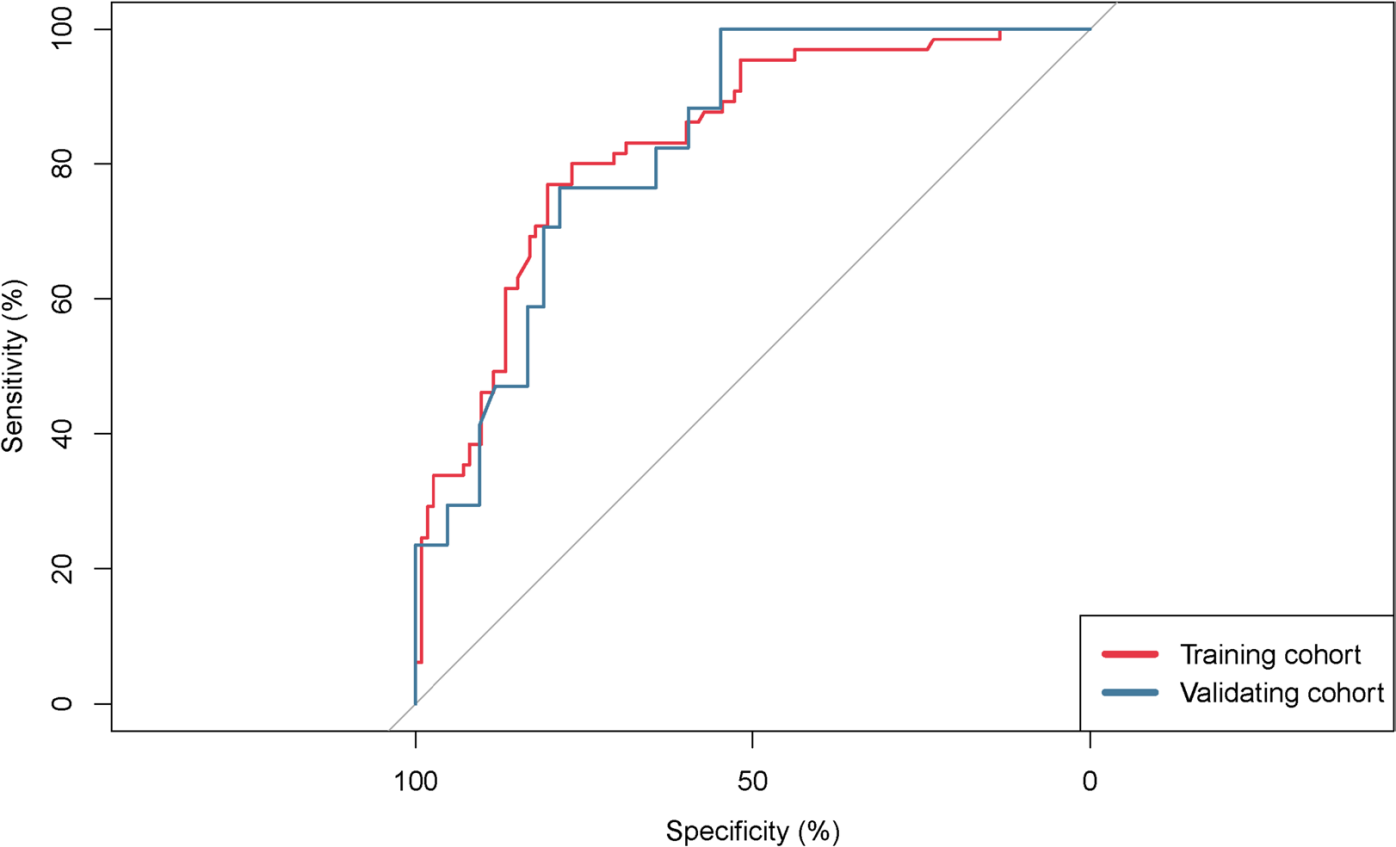
The ROC curve for using the nomogram to predict PONV in the training cohort (*n* = 177) and validation cohort (*n* = 59). PONV, postoperative nausea and vomiting; ROC, receiver operating characteristic

**Fig. 4:**
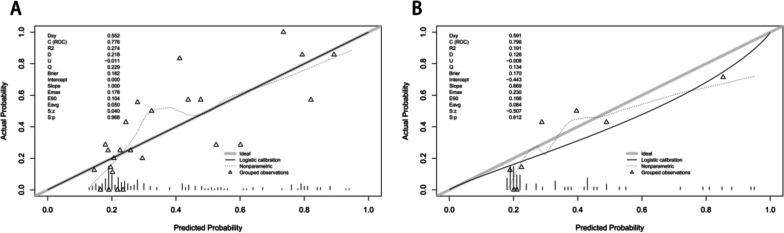
**A** The calibration curve of the nomogram for evaluating PONV risk in the training cohort (*n* = 177). **B** The calibration curve of the nomogram for evaluating PONV risk in the validating cohort (*n* = 59). The horizontal axis of the calibration curve represents the predicted probability of PONV calculated by the nomogram, and the vertical axis represents the actual probability of PONV. The light blue line through the origin represents the ideal diagnosis result, and the black solid line represents the prediction result of this model. The closer the prediction solid line of the model is to the ideal diagnosis result, the better the prediction efficiency of the model. PONV, postoperative nausea and vomiting

**Fig. 5:**
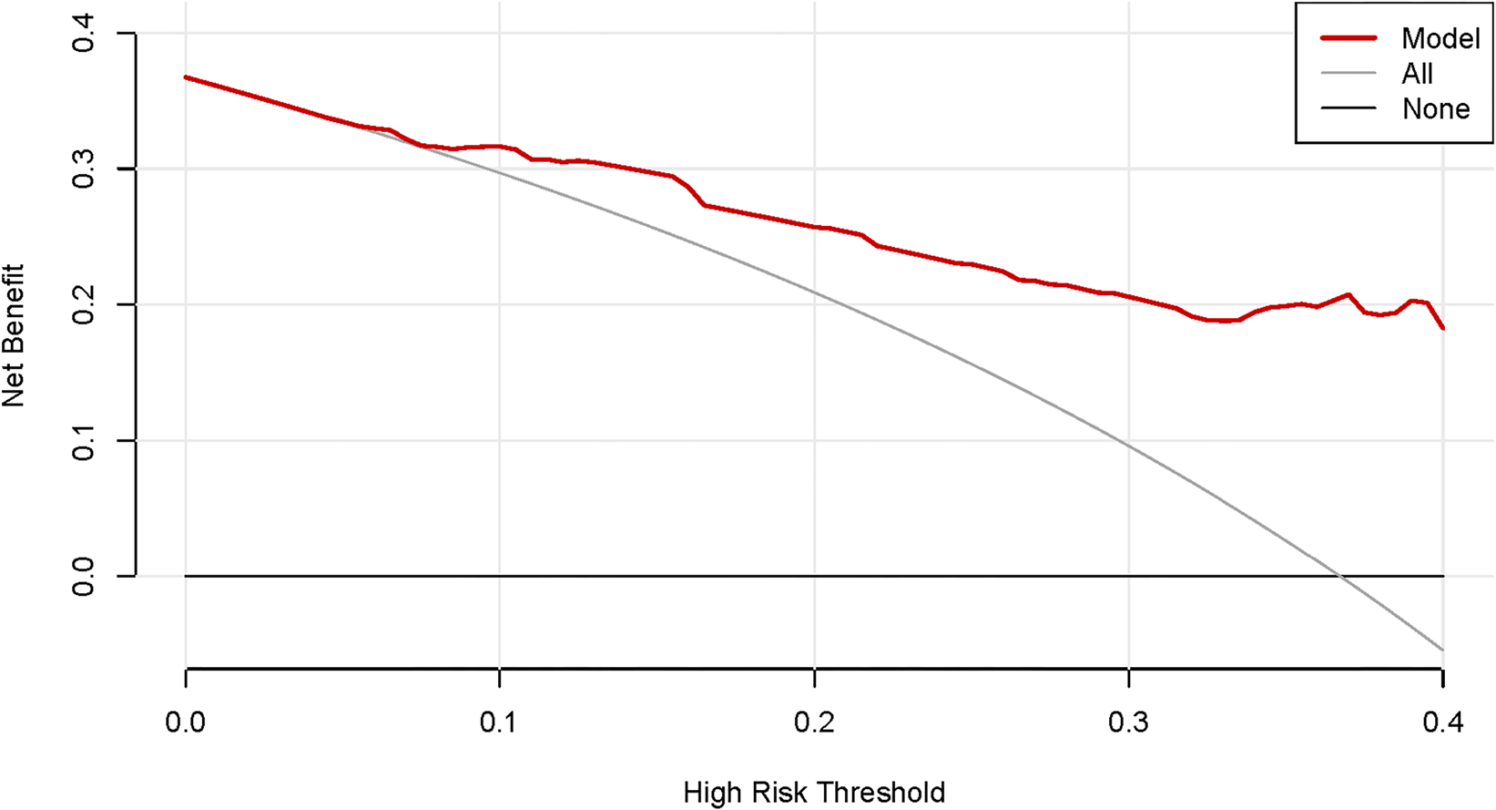
DCA of the nomogram for predicting PONV in adult patients undergoing emergency surgery. The horizontal axis represents the domain probability value, and the vertical axis represents the net benefit rate. The light blue curve represents the assumption that PONV occurs in all adult emergency surgery patients. The black line represents the assumption that no PONV occurs in all adult emergency surgery patients. The red curve represents the nomogram constructed in this study. When the probability range of the domain is > 8%, the model has a high benefit for the prediction of PONV. DCA, decision curve analysis; PONV, postoperative nausea and vomiting

**Fig. 6:**
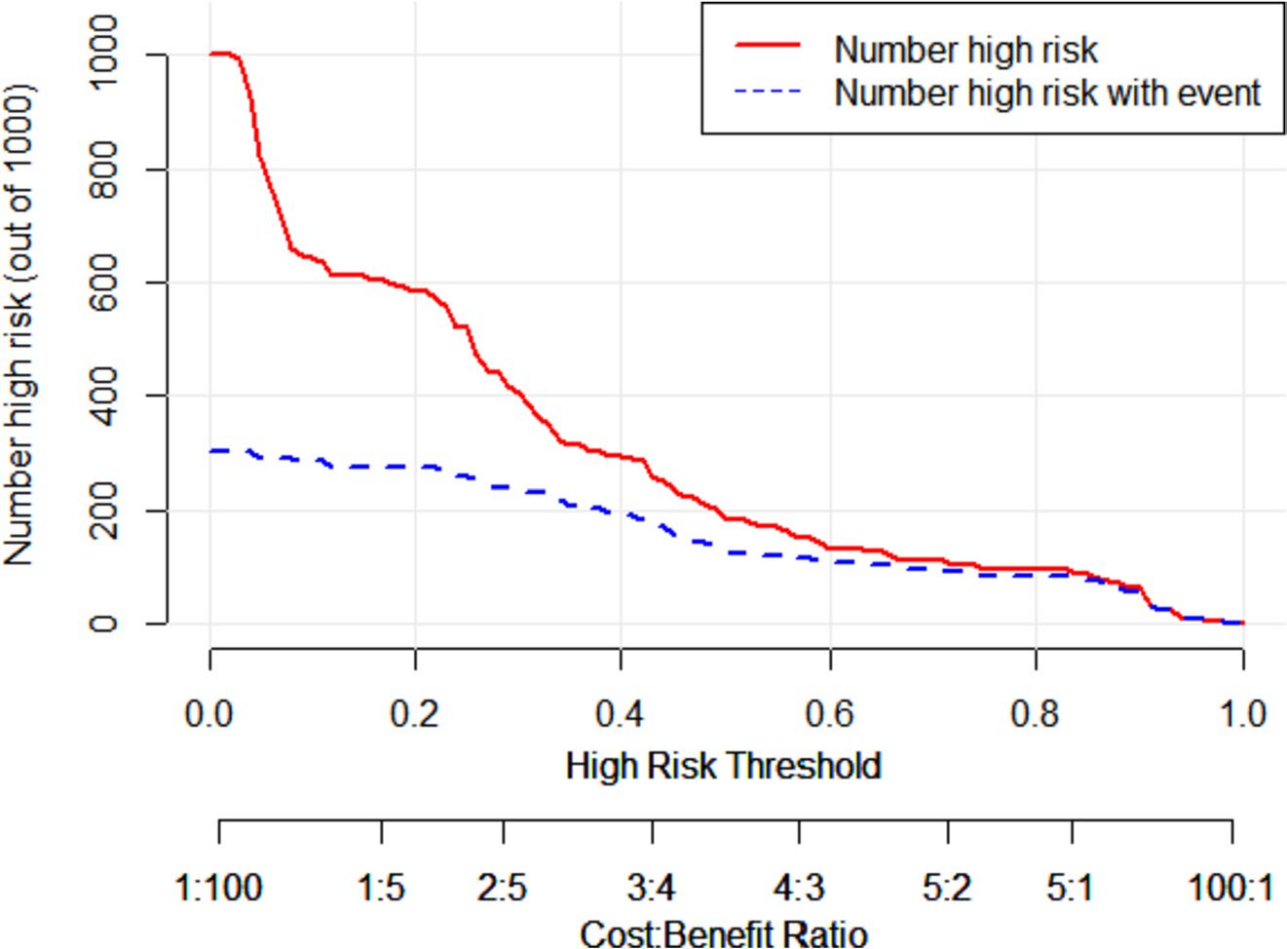
Clinical impact curve (CIC) of nomogram. The y-axis represents the number of high-risk individuals classified by the model at each threshold probability, assuming there are 1000 patients. The red curve (Number high-risk) represents the number of individuals classified as high-risk at each threshold probability by the model. The blue dashed line (Number high-risk with event) represents the actual number of high-risk individuals at each threshold probability

These independent risk factors were used to form a PONV risk estimation nomogram by the rms package of R version 4.2.2 (Fig. 2). Different score values were set according to the different OR values of each factor. Then according to the score value of each factor, the corresponding position on the horizontal axis was used to obtain the score of the factor. The score of each factor was summed to obtain the total score. The total score corresponded to the point on the PONV risk axis (i.e., the probability value for the occurrence of PONV in patients). The nomogram demonstrated good accuracy in estimating the risk of PONV, with an area under the ROC curve of 0.832 (95% CI, 0.771–0.893) in the training cohort and 0.827 (95% CI, 0.722–0.932) in the validation cohort (Fig. 3). In addition, calibration curves showed good agreement on the occurrence of PONV between the risk estimation by the nomogram and the actual occurrence (Fig. 4). DCA was used to evaluate the clinical practicability of the nomogram. The results showed that when the domain probability of the nomogram was > 8%, the benefit was higher and the nomogram domain selection probability range was larger, indicating that the clinical practicability was strong (Fig. 5). The CIC showed that the "number high risk" line and the "number high risk with event" lines are relatively close to each other, indicating that using this nomogram model for predicting PONV in emergency surgery patients leads to a great clinical net benefit (Fig. 6).

### Risk of PONV based on the nomogram scores

The Hosmer–Lemeshow goodness of fit test coefficient of the nomogram was 0.212. The C statistic and optimal cut-off probability were 0.832 and 0.393, respectively, in the training cohort and 0.827 and 0.364, respectively, in the validation cohort (Table 4).

**Table 4:** Accuracy of the nomogram in estimating the risk of PONV

Variable	Training cohort	Validating cohort
Area under the ROC curve, concordance index	0.832 (0.771–0.893)	0.827 (0.722–0.932)
Hosmer–Lemeshow test	0.212	
Cut-off score	102.813	97.882
Youden index	0.572	0.550
Sensitivity, %	0.769	0.765
Specificity, %	0.804	0.786
Positive predictive value, %	0.694	0.591
Negative predictive value, %	0.857	0.892
Positive likelihood ratio	3.916	3.569
Negative likelihood ratio	0.287	0.299

*PONV* Postoperative nausea and vomiting, *ROC* Receiver operating characteristic

## Discussion

PONV poses a tremendous challenge to postsurgical recovery, as accidental aspiration may endanger the patient’s life. In this study, 87 patients had PONV, accounting for 36.86% of the total 236 participants. We conducted a detailed exploration of PONV susceptibility factors based on patient characteristics, surgical and anaesthesia factors, as well as preoperative ultrasound exploration. The results indicated that female sex, PONV history, migraine history, and CSA were independent risk factors for PONV. These four factors were used to build the prediction model, and a nomogram was constructed to facilitate its visual and practical application. After the model was established, it was evaluated and verified from multiple perspectives via the area under the ROC curve, calibration curve, DCA and CIC.

Among the included indicators, females, PONV history and migraine history have been widely confirmed to have a high predictive value for PONV. Apfel et al. [9] and Koivaranta et al. [10] included these factors in their own respective studies to construct PONV prediction models, which have been widely used in clinical practice. However, the Koivuranta model includes children, and it has now been verified that the main risk factors for PONV in children are not the same as in adults [22]. While the Apfel model only applies to adults, fentanyl, alfentanil, isoflurane, enflurane and sevoflurane were mainly used for anaesthesia induction at the time that the study was conducted [9]; these drugs would have been more likely to cause PONV compared with propofol, which is currently more commonly used [6]. In addition, the predictive efficacy of these two scores is low, as they are only based on the patients’ congenital conditions [14].

A major difference between patients undergoing emergency surgery and those undergoing elective surgery is that they often lack adequate bowel preparation [23]. In this study, preoperative ultrasound assessment was combined with patient, surgical and anaesthesia factors to build a multidisciplinary prediction model. Previous studies mainly explored the relationship between preoperative gastric volume or average gastric volume and PONV [14]. We considered that the volume and average gastric volume were essentially obtained using CSA, age, and weight through a purely mathematical calculation; the differences between age and CSA in univariate regression analysis were statistically significant, and the preoperative preparation time of emergency patients was relatively short. As it was more practical to save the time required for calculating the stomach volume, CSA was chosen to replace stomach volume and average stomach volume in our calculations. Furthermore, both DCA and CIC demonstrate that the clinical utility of this nomogram model is robust. Compared with empirical medication, using bedside ultrasound examination of the gastric antrum in preoperative emergency patients and then applying this nomogram model allows for a more accurate identification of high-risk patients. It also offers a relatively wide range of domain probabilities and a higher clinical net benefit rate.

In this study, the established prediction model was evaluated from multiple perspectives, and the reliability of the model was demonstrated. The presentation of the model in the form of a nomogram is more intuitive, flexible, and easy to apply for medical staff who need to quickly identify emergency operation patients at risk of PONV and implement therapeutic measures (including preoperative prophylactic application of antiemetic drugs, perioperative application of dexmedetomidine and administration of auxiliary oxygen) to improve postoperative recovery.

Some limitations are acknowledged in the present study. First, this was a single-centre study. The number of samples included was relatively small, and the model has not been verified externally. Further studies using larger sample sizes across multiple centres are needed. Second, the included imaging and clinical laboratory examination items were limited; therefore, some known or unknown risk factors related to PONV were not accounted for in our analysis. Subsequent studies can further expand patient data on the basis of this study and screen for indicators with a higher correlation with PONV.

In conclusion, four independent risk factors for PONV identified via multivariate regression analysis were combined to construct a nomogram to predict PONV. This nomogram can enhance preoperative assessment by predicting the risk of PONV in adult patients before emergency surgery.

## Data Availability

The data that support the findings of this study are available from the corresponding author, [XZ], upon reasonable request.

## Abbreviations

PONV: Postoperative nausea and vomiting
DCA: Decision curve analysis
CIC: Clinical impact curve
AP: Anteroposterior diameter
CC: Craniocaudal diameter
CSA: Cross-sectional area
BMI: Body mass index
ICC: Intraclass correlation coefficient
ROC: Receiver operating characteristic
